# Circulating tumor DNA-guided treatment decision in metastatic castration-resistant prostate cancer patients: a cost-effectiveness analysis

**DOI:** 10.1177/17588359241305084

**Published:** 2024-12-15

**Authors:** Catharina J. P. Op ’t Hoog, Sabien J. E. Bosman, Emmy Boerrigter, Niven Mehra, Inge M. van Oort, Nielka P. van Erp, Wietske Kievit

**Affiliations:** Department of Pharmacy, Research Institute for Medical Innovation, Radboud University Medical Center, Nijmegen, The Netherlands; IQ Health, Research Institute for Medical Innovation, Radboud University Medical Centre, Nijmegen, The Netherlands; Department of Pharmacy, Research Institute for Medical Innovation, Radboud University Medical Center, Nijmegen, The Netherlands; Department of Medical Oncology, Research Institute for Medical Innovation, Radboud University Medical Center, Nijmegen, The Netherlands; Department of Urology, Research Institute for Medical Innovation, Radboud University Medical Center, Nijmegen, The Netherlands; Department of Pharmacy, Research Institute for Medical Innovation, Radboud University Medical Center, Nijmegen, The Netherlands; IQ Health (160), Research Institute for Medical Innovation, Radboud University Medical Center, P.O. Box 9101, Nijmegen 6500 HB, The Netherlands; IQ Health, Research Institute for Medical Innovation, Radboud University Medical Center, Nijmegen, The Netherlands

**Keywords:** androgen receptor pathway inhibitors, biomarkers, chemotherapy, circulating tumor DNA (ctDNA), cost-effectiveness analysis, health services research, hormone therapy, liquid biopsy, prostate cancer

## Abstract

**Background:**

The androgen receptor pathway inhibitors (ARPI), abiraterone acetate and enzalutamide, are commonly used in first-line treatment of patients with metastatic castration-resistant prostate cancer (mCRPC). However, early resistance to ARPI treatment occurs frequently. Traditionally, the response is evaluated 3–6 months after the start of treatment. However, recent findings indicate that by detecting circulating tumor DNA (ctDNA) at baseline and 4 weeks after ARPI treatment initiation, patients with a nondurable response can be identified after 4 weeks of treatment, enabling an early switch to alternative treatments.

**Objective:**

This study aims to evaluate the cost-effectiveness of ctDNA-guided treatment switch after 4 weeks of ARPI therapy in mCRPC patients compared to standard of care.

**Design:**

A cost-effectiveness analysis.

**Methods:**

A cost-effectiveness analysis was conducted by creating a Markov state transition model to simulate progression, mortality, and treatment costs over a 5-year time horizon comparing ctDNA-guided care versus standard of care. The outcomes measured were incremental treatment costs per life-years and quality-adjusted life-years (QALYs) gained.

**Results:**

The analysis showed an incremental cost-effectiveness ratio of €65,400.86 per QALY gained and an incremental net monetary benefit of €2716.62. Thereby, the use of ctDNA-guided treatment was cost-effective in comparison to standard care in 74% of the simulations using a willingness-to-pay threshold of €80,000 per QALY gained.

**Conclusion:**

Our study demonstrated the cost-effectiveness of using a ctDNA-guided early therapy switch in non-responders after only 4 weeks of first-line ARPI therapy in mCRPC patients. This paves the way for implementing ctDNA-guided treatment decisions in clinical practice.

## Introduction

Treatment with the androgen receptor pathway inhibitors (ARPI), abiraterone acetate and enzalutamide, has been approved for the treatment of several stages of prostate cancer, including metastatic castration-resistant prostate cancer (mCRPC) and metastatic hormone-sensitive prostate cancer (mHSPC).^1,2^ While these potent ARPIs have led to important survival benefits, they also increase healthcare costs. Furthermore, all patients will eventually develop resistance to ARPIs over time.^3–7^ Approximately 20%–30% of mCRPC patients treated with first-line ARPIs show intrinsic resistance or develop resistance shortly after the start of treatment, often within 6 months.^3,4,8^ Currently, treatment response assessment relies on biochemical, radiographic, and clinical evaluations guided by the RECIST 1.1 and Prostate Cancer Clinical Trials Working Group 3 (PCWG3) criteria.^9,10^ Assessment before approximately 3 months of therapy is considered unreliable due to early fluctuations in serum prostate-specific antigen and tumor flare which disturbs radiographic evaluation.^9^ Confirmation of disease progression in patients with non-RECIST 1.1 evaluable disease is done according to the PCWG3 criteria, which can take up to 6 months.^10^ Consequently, patients who experience no or limited benefit from ARPI treatment will be identified after 3–6 months of treatment, when imaging shows radiographic progression. Radiographic progression is often accompanied by clinical progression. The delay in treatment response evaluation hinders the timely switch to more effective drugs when patients are still in a more favorable condition. This is while multiple alternative treatment options are currently available.^11,12^

Several studies have shown the potential of plasma circulation tumor DNA (ctDNA) as a prognostic biomarker for the survival of patients with mCRPC, for personalizing treatment, and for predicting treatment response.^13–17^ Recently, Tolmeijer et al. demonstrated that early changes in ctDNA fraction were strongly linked to the duration of first-line ARPI treatment benefit and survival in patients with mCRPC. Patients with progression within 6 months after treatment initiation, that is, non-durable response, could successfully be identified after only 4 weeks of first-line ARPI treatment by measuring detectable baseline and 4-week on-treatment ctDNA fraction.^18^ Consequently, measurement of early changes in ctDNA fraction may improve the management of mCRPC by supporting early therapy switch or intensification in patients unlikely to experience durable treatment response.^18^ Though it is currently unknown what the financial consequences are of the use of ctDNA measurements, which increases medical costs per patient. On the contrary, monitoring of ctDNA could facilitate early treatment switch in non-responders and thereby potentially reduce ARPI drug costs while increasing survival and quality of life. Yet, it is unclear whether the additional costs that are introduced by ctDNA measurements compromise the benefits.

This study describes the preplanned cost-effectiveness analysis comparing the outcomes of the early ctDNA-guided treatment switch after 4 weeks of ARPI therapy, as described by Tolmeijer et al., to the standard of care approach, where treatment decisions are made after 6 months of therapy.

## Methods

### General considerations

To determine the potential cost-effectiveness of using ctDNA as an early response marker for switching therapy after 4 weeks of treatment, a Markov state transition model has been developed. The costs, effects of the intervention arm, ctDNA-guided treatment, and the standard of care arm have been modeled over a time horizon of 5 years at 3-week cycles after the initial cycle of 4 weeks. The analysis was performed from a Dutch healthcare perspective. The main comparison is the effect of the early ctDNA-guided switch to second-line therapy after 4 weeks of ARPIs on costs, life-year, and quality-adjusted life-year (QALY) in non-responders compared to switch after 6 months (usual care) of therapy. The use of ctDNA introduces a different duration of first-line ARPI treatment of mCRPC patients and allows patients to switch to a different type of treatment (i.e., taxane-based chemotherapy) or best supportive care. Alternative treatment options, such as PARP inhibitors and immunotherapy, were not available at the time of analysis and therefore not incorporated into the model. The ctDNA measurement will result in higher costs and a prolonged time to progression when it facilitates an earlier switch from an ineffective treatment to a possibly effective treatment. Early treatment decisions will also lower the drug costs of ARPI treatment. This study is conducted in adherence with the Dutch guidelines for economic analyses and in the writing of this report we followed the Consolidated Health Economic Evaluation Reporting Standards statement, which can be found in Supplemental File 4.^19–21^

### Model overview

**Figure 1.**
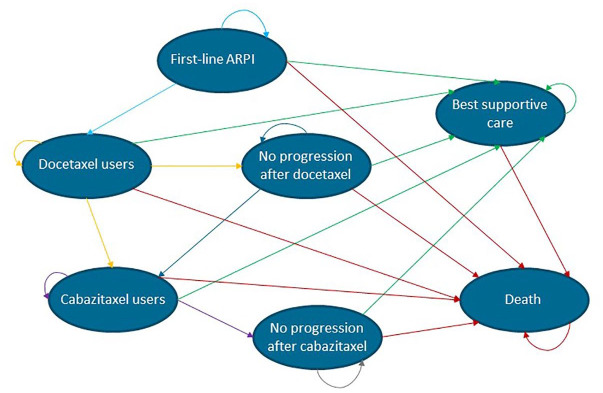
Schematic overview of the Markov model in mCRPC patients. mCRPC, metastatic castration-resistant prostate cancer.

The Markov model consisted of seven health states: first-line ARPI treatment (enzalutamide or abiraterone acetate), second-line docetaxel, third-line cabazitaxel, no progression after docetaxel, no progression after cabazitaxel, best supportive care, and death (Figure 1). All patients entered the model in the first-line ARPI treatment health state. Once patients’ disease progresses on ARPI treatment, they continue with second-line docetaxel. It is assumed that a switch to second-line treatment occurs after 6 months at the earliest in standard of care. After disease progression on docetaxel, patients start with third-line cabazitaxel. Since docetaxel and cabazitaxel are given for a usual amount of 10 cycles, 10 sub-states have been incorporated into the model to incorporate the time-dependency of these medications into the model. Patients entered the docetaxel/cabazitaxel state in the first sub-state. When they did not progress or die, they progressed to the second sub-state, and so on. After 10 cycles of either docetaxel or cabazitaxel, patients with no progression stay in the no progression after docetaxel/cabazitaxel health states until progression. Patients can move from every health state to the best supportive care or death.

The model structure was the same for both strategies that were compared, with the exception that patients in the ctDNA-guided treatment arm can switch to second-line treatment with docetaxel after 4 weeks of therapy instead of 6 months when no response is expected based on ctDNA measurements. After the ctDNA-guided switch, patients are treated the same as in the standard of care.

The model includes a fictitious cohort of 1000 patients with mCRPC patients starting the first line of treatment with abiraterone acetate or enzalutamide. Perfect adherence to the guidelines and the treatment is assumed for each fictitious patient. Primary outcomes included healthcare costs and QALYs, which were used to calculate the incremental cost-effectiveness ratios (ICERs). Costs and QALYs were accumulated over a 5-year horizon at 3-week cycles after the first cycle of 4 weeks. Costs and utilities are discounted each year at 4% and 1.5%, respectively, in agreement with our national guidelines for pharmaco-economic research.^22^ The model was created using Microsoft Excel^®^ (Microsoft Corporation, Redmond, WA, USA).

### Model data input

A complete list of all input parameters can be found in Supplemental File 1. The assumption was made that patients had a probability of 0.5 to receive either abiraterone acetate or enzalutamide in first-line treatment. To determine the time-dependent transition probabilities between health states of progression-free survival (PFS) and overall survival (OS), data from the different treatments defined in our model was used. In the model, PFS and OS data from the registration trials were used,^3,4,23–25^ except for docetaxel where more recent survival data from the FIRSTANA trial are used as the treatment landscape has changed since its approval.^26^ The selection of the clinical trials for PFS and OS data is found in Supplemental File 3. Progression and mortality data for best supportive care and death were extracted from the literature and reports of the Dutch National Health Care Institute.^27–29^ For the intervention arm, the proportion of patients who exhibited detected ctDNA levels both at baseline and after 4 weeks of treatment and subsequently experienced a non-durable response was 0.85 based on the findings of Tolmeijer et al.^18^ Since ctDNA can differentiate between both patients with non-durable and patients with durable response on first-line ARPI, patients with durable response have a longer PFS and OS than the median in the registration trials. Therefore, PFS and OS data from the Tolmeijer et al. study were used in the intervention arm to model PFS and OS in the patients with the predicted durable response on first-line ARPI.^18^

The probabilities of experiencing adverse events (AEs) were derived from the literature.^30,31^ For each treatment group, the most common (frequent occurrence) and severe adverse events (grade ⩾3) have been included since these are expensive to treat.

Prices of the medication were drawn from the Dutch National Health Care Institute^32^ and are based on the drug dosages as described in the clinical guidelines for prostate cancer and the drug labels.^2^ A mean body surface area was used to calculate the dose of docetaxel and cabazitaxel.^33^ The costs of ctDNA analysis were €350 per sample, requiring two samples per patient (e.g., baseline and at 4 weeks), and including one additional sample per patient for resolving germline variants from putative somatic mutations and to exclude somatic mutations present in white blood cell DNA. Costs of hospital visits, diagnostic tools, best supportive care, and adverse events were extracted from the literature.^22,34,35^ All costs were inflated to 2022 in euros using the Dutch consumer price index.^36^

Utility weights for the treatment groups used in this model were estimated based on the functional assessment of the cancer therapy-prostate (FACT-P) questionnaire as reported in clinical trials.^37–40^ A published and formerly utilized model was used to convert FACT-P to EuroQoL-5D (EQ-5D) utility values as the preferred generic health-related quality of life value. The used model has been validated for mapping EQ-5D index scores from FACT-P in mCRPC patients and was found to have a good predictive ability.^41^ The utility weight for best supportive care was derived from the literature.^42^

### Analysis

The model analyses ICER, expressed in extra costs that need to be paid per QALY gained for the ctDNA-guided treatment compared to usual care. A Monte Carlo simulation was run using 5000 iterations to generate the mean incremental cost-effectiveness (ICE) with 95% confidence intervals. With every iteration, a random value of the parameters was drawn from the β- and γ-distributions that were used for probabilities, utilities, and costs, respectively. Plausible uncertainty was taken into account for all parameter range distributions. The results of the Monte Carlo simulation are graphically represented in a cost-effectiveness plane and an acceptability curve. As a willingness-to-pay (WTP) threshold, we used the value of €80,000 per QALY gained which is recommended for use in the context of the Dutch healthcare system if the disease has a high disease burden which is deemed to be the case as a metastatic disease according to the Dutch guidelines.^43,44^ ICERs below this value are considered to be cost-effective.

### Sensitivity analyses

Currently, enzalutamide is patented by Astellas under the name Xtandi^®^. This patent is set to expire in the near future, hypothetically resulting in significant changes in the pricing of this drug. The patent of abiraterone acetate, cabazitaxel, and docetaxel has already expired and generic drug formulations are used. To determine the effect of decreased drug pricing on cost-effectiveness, decreased drug costs of 90% were used as agreed with experts. It is also expected that the costs of ctDNA analyses will decrease. A reduction of the ctDNA analysis costs by up to €100 was deemed realistic.

Other sensitivity analyses performed are variations in the proportion of patients that receive enzalutamide or abiraterone acetate (varying between 0 and 1), as well as changes in the efficacy of the prediction value of ctDNA (varying between 0.65 and 0.95).

We analyzed the incremental net monetary benefit (iNMB) as the outcome of the sensitivity analyses. The iNMB combines both the effect of the intervention on quality of life multiplied by the WTP value and the costs in a single number. A positive iNMB indicates that the intervention is preferable over the usual care at the given WTP threshold. The results of the sensitivity analyses are represented in a Tornado diagram.

### Scenario analyses

Four scenarios have been evaluated in the model to explore the effect of certain assumptions and model choices on the conclusions of this study. The base model uses PFS and OS data based on results from the registration trials. ctDNA can differentiate between patients with non-durable responses and patients with durable responses on first-line ARPI. Patients with durable responses have a longer PFS and OS than the median in the registration trials. The data on PFS and OS from the study of Tolmeijer et al.^18^ have been used in the intervention arm. However, Tolmeijer et al. only included 81 patients, and data might therefore be less representative. Therefore, the same PFS and OS data for enzalutamide and abiraterone as the standard of care arm are used in the first scenario analysis.

For the second scenario, the data of the PROSELICA trial for cabazitaxel have been used as progression and mortality data for patients who switch to docetaxel after 4 weeks of therapy in the intervention arm instead of the CARD trial in the base model. In the CARD trial, cabazitaxel is used as third-line therapy.^24^ In the PROSELICA trial, cabazitaxel is used as second-line therapy. Arguably, cabazitaxel can be seen as second-line therapy in patients who switch to docetaxel after 4 weeks of ARPI therapy.^24,45^

In the third scenario, patients who switch to docetaxel after 4 weeks of therapy get a higher utility in the intervention arm, because it is expected that the quality of life in these patients does not decrease as fast. A mean utility of abiraterone and enzalutamide is used as a utility for docetaxel in the intervention arm.^37–39^

In the base model, progression and mortality data for docetaxel are extracted from the FIRSTANA trial.^26^ In this trial, docetaxel is given as first-line therapy. In the standard of care arm of this model, docetaxel is given as second-line therapy. In current literature, no studies have been performed where docetaxel is used as a second-line treatment after abiraterone or enzalutamide. Only real-life data from Japan are available. Therefore, the Japanese data are used in the last scenario analysis of the model.^46^

## Results

### Base-case analysis

**Figure 2.**
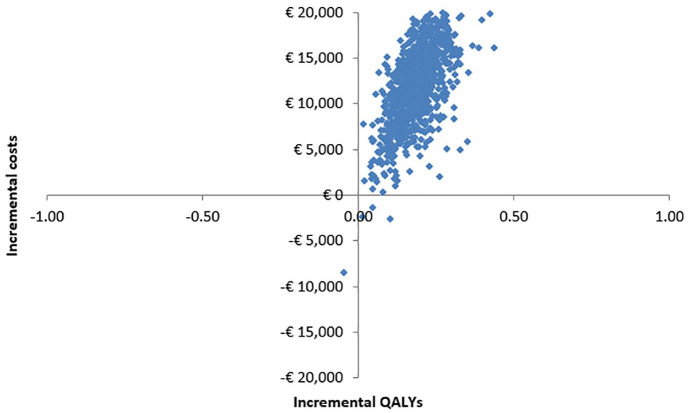
ICE plane of the base model. ICE, incremental cost-effectiveness.

**Figure 3.**
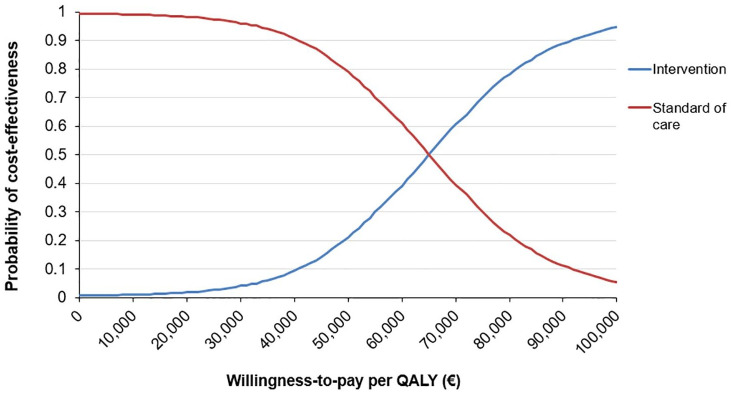
Cost-effectiveness acceptability curve of the base model.

Based on our model, it is estimated that over a 5-year time horizon, the ctDNA-guided strategy will result in a gain of 0.186 QALYs against an increase in costs of €12,169. The ICER is estimated to be €65,400.86 per QALY gained and the iNMB €2716.62, with 74% of the simulations being lower than the WTP threshold of €80,000 per QALY gained. The results of the Monte Carlo simulation are shown in Table 1. The ICE plane and the cost-effectiveness acceptability curve are shown in Figures 2 and 3, respectively.

**Table 1.** Results of the different scenarios concerning costs and QALY’s related to the ctDNA-guided strategy or standard of care and the percentage of cost-effectiveness based on a WTP threshold of €80,000.

Analysis	ctDNA-guided strategy		Standard of care		ICER	Cost-effective, %
	Costs, €	QALY	Costs, €	QALY		
Base case	€124,886.28 (€115,282.09–€133,873.52)	1.817 (1.491–2.111)	€112,716.43 (€107,543.27–€118,146.29)	1.631 (1.353–1.866)	€65,400.86	74
PFS and OS data registration trials	€113,819 (€108,382–€119,340)	1.62 (1.33–1.85)	€112,696 (€107,315–€118,204)	1.63 (1.34–1.87)	€90,845.00	3
PROSELICA trial	€132,192 (€121,379–€142,404)	1.81 (1.48–2.11)	€120,009 (€114,019–€126,302)	1.63 (1.35–1.87)	€67,406.81	65
Utility docetaxel	€124,968 (€115,096–€133,906)	1.82 (1.48–2.12)	€112,776 (€107,394–€118,174)	1.63 (1.35–1.86)	€63,947.32	76
Real-life data docetaxel	€125,068 (€115,415–€134,267)	1.85 (1.51–2.15)	€111,660 (€105,462–€117,635)	1.65 (1.37–1.90)	€67,511.14	73

ctDNA, circulating tumor DNA; ICER, incremental cost-effectiveness ratio; OS, overall survival; PFS, progression-free survival; QALY, quality-adjusted life years; WTP, willingness-to-pay.

### Sensitivity analysis

**Figure 4.**
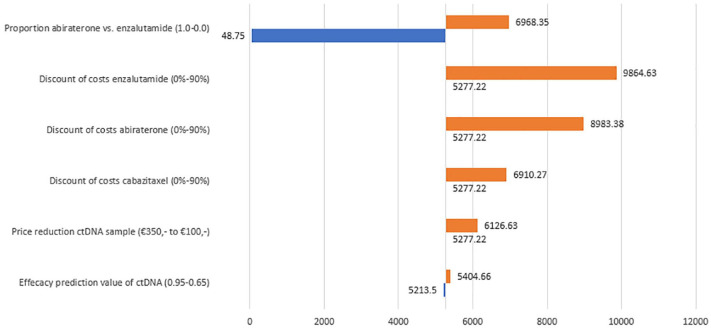
Tornado diagram of the sensitivity analysis. *X*-axis: iNMB. iNMB, incremental net monetary benefit.

Sensitivity analyses were performed with variations in the proportion of patients that use abiraterone or enzalutamide, price reduction of enzalutamide, abiraterone, and cabazitaxel, the effectiveness of the predictive value of ctDNA, and the cost reduction for the ctDNA measurement. In the Tornado diagram, as shown in Figure 4, it is shown that the proportion of abiraterone and enzalutamide users has the largest effect on the iNMB. When only abiraterone is used the iNMB drops to €49, which is however still cost-effective. When prices of the different drugs will drop in the future, the iNMB will increase further.

### Scenario analysis

In the intervention arm, PFS and OS data from the study of Tolmeijer et al. are used. In the first scenario analysis, the same PFS and OS data for enzalutamide and abiraterone are used in the intervention arm as in the standard-of-care arm. In this case, the intervention is cost-effective in 3% of the patients and an iNMB of −€1867.97. This shows that the source of the progression and mortality data of abiraterone and enzalutamide have a large impact on the results of the model.

When using the PFS and OS of cabazitaxel data from the PROSELICA trial instead of the CARD trial, the intervention arm is cost-effective in 69% of the patients with an iNMB of €5058.16.

In the scenario where patients who switch to docetaxel after 4 weeks in the intervention arm are given a higher utility, the intervention is cost-effective in 76% of the patients with an iNMB of €5495.08.

If the real-life data from Japan is used as a source for the progression and mortality data in second-line docetaxel treatment for both the standard-of-care arm as the intervention arm, the intervention is cost-effective in 73% of the patients with an iNMB of €4809.29. The results of the scenario analyses are shown in Table 1.

## Discussion

To our knowledge, this study is the first to evaluate the ICE of ctDNA-guided treatment decisions in comparison to conventional response assessment in first-line ARPI-treated mCRPC patients. Importantly, ctDNA collection and measurement is relatively easy to perform and is increasingly reported as a pragmatic strategy to improve patient management.^47^ Although it comes with extra costs, our results suggest that the use of ctDNA can be cost-effective with a mean ICER of €65,400.86 per QALY and a 74% chance of being cost-effective given a WTP threshold of €80,000, which is in the Netherlands generally used for patients with a high burden of disease such as mCRPC.^15,18,48^

The use of ctDNA analyses enables clinical decision-making short after the start of treatment and could facilitate early treatment switch to potential other life-prolonging therapies in patients with mCRPC as demonstrated in the study of Tolmeijer et al. Early therapy switches in patients with a non-durable response can prevent patients from receiving ineffective and costly drugs.^18^ Even when no further treatment options are available, early discontinuation of an ineffective therapy based on ctDNA measurements may reduce patient exposure to unnecessary drug-related AEs leading to improved quality of life and potential costly toxicity management.

Our sensitivity analysis showed no large effect on the iNMB when price reductions were applied in treatment options or ctDNA analysis. The patent of enzalutamide will expire in 2028 in Europe,^49^ and it is expected that generic drug formulations will be available in the near future. Since changes in drug costs have been taken into account in our analysis, the results of our model will still be relevant to generic drug formulations. In the current situation with higher costs of enzalutamide, changes in the proportion of patients that use abiraterone or enzalutamide do have a considerable impact on cost-effectiveness which is the result of the lower costs of abiraterone. As of now, the proportion of abiraterone users has increased due to the generic formulation and consequently lower costs in the Netherlands. Recently, many patients have received treatment intensification when diagnosed with high-risk localized and metastatic hormone-sensitive prostate cancer, and therefore will not be eligible for ARPIs when developing castration resistance. In addition, combination regimens have been approved for mCRPC consisting of PARP inhibitors in combination with ARPIs, for those patients treated “upfront” with androgen deprivation therapy (ADT) only or ADT in combination with docetaxel.^50–52^ The rapidly changing landscape of therapy lines makes it hard to predict the proportion of patients that will use abiraterone and enzalutamide monotherapy for mCRPC setting, but this likely will decrease due to migration of these drugs to earlier settings or combination regimens with ARPIs that are introduced.

In our study, scenario analyses were used to explore the effect of certain assumptions and model choices on our results. The scenario analyses showed minimal differences in cost-effectiveness, except for the source of the data used to estimate the PFS and OS in patients with a durable response. In this scenario, the use of ctDNA would likely not be cost-effective. This gives a level of uncertainty in our analysis, as the source of data has a large impact. However, in general, PFS and OS data that are derived from registration trials, which were conducted in a randomized-controlled setting and larger patient populations, make the PFS and OS outcomes robust. However, the median PFS and OS data reported in these trials are based both on durable and non-durable responders. The relatively small study of Tolmeijer et al. is the only study thus far that presents survival outcomes separately for both groups and therefore the data of the patients with durable responses was used in our analysis. Larger studies reporting PFS and OS data under ctDNA-guided therapy would make the model more accurate since patients that are potentially non-durable responders can be differentiated early after the start of treatment and thus make the survival of patients that do benefit from treatment potentially longer. However, studies investigating the predictive value of ctDNA on mCRPC patients to evaluate treatment response are lacking. As of now, only studies that highlight the use of ctDNA as a prognostic factor or for decision-making in therapy choice are available.^17,53,54^

Our analysis has several limitations, and the results need to be interpreted within the context of the assumptions made in the model. The study was conducted before several phase III clinical trials and reported survival benefits with the use of ARPI in less advanced disease states, such as mHSPC.^5–7^ Therefore, the used setting in our model with ARPI-naïve mCRPC patients might be less representative of currently treated patients progressing to mCRPC. Furthermore, newer lines of therapy, such as PARP inhibitors, radioligand therapy, and immunotherapy, have become available. In our analysis, only docetaxel and cabazitaxel were used as alternative treatments. Our analysis does not include these new therapies and their additional costs. It is unknown what the cost-effectiveness is when switching to a more expensive therapy or when therapy is added to ARPI (e.g., PARP inhibitors) instead of switching to taxane-based chemotherapy. Still, it is expected that ctDNA-guided treatment optimization is a treatment-unrelated marker that can be used to reveal tumor response to any line of treatment. We hypothesize that the results of the model therefore remain relevant for a large subset of mCRPC patients.

Moreover, several factors such as comorbidities or other treatments could be important covariates that contribute to uncertainty in the data. The analysis included only those AEs with relatively frequent occurrence and that are expensive to treat. Low-grade AEs and AEs with low frequency were not included in the analyses but might impact healthcare costs.

In addition, key parameters, such as PFS and OS data in the analysis, were derived from previously published literature with a limited follow-up and in a controlled clinical trial setting, which may affect the outcomes of our analyses. Longer follow-up data may adjust toxicity and survival outcomes when patients cannot stay on treatment continuously as a result of long-term, low-grade toxicity. Furthermore, the real-world population might experience other treatment benefits of ARPIs than reported in the registration studies. A cost-effectiveness analysis of ctDNA guidance in consecutive treatment lines based on a real-world population with a lifetime follow-up may be more representative. However, these trials are costly and require a long follow-up time. To assess the cost-effectiveness without relying on such trials, our model can provide reliable predictions based on the currently available data. Furthermore, we have chosen a time horizon of 5 years. It would be preferable to choose a lifetime horizon. However, as shown in the registration trials, the median OS in mCRPC patients is approximately 1–3 years at the time of analysis,^3,4,23–25^ When choosing a lifetime horizon, several assumptions have to be made that are difficult to justify. Since the follow-up time of the clinical trials is often 5 years maximum, data are lacking. We therefore have chosen a 5-year time horizon. In addition, the model is based on an ideal situation, where adherence to treatment and following the guidelines is 100%. It is also assumed that the advice to switch to second-line treatment after 1 month based on the change in ctDNA fraction is always followed which might not be realistic and may lead to an overestimation of the cost-effectiveness. On the one hand, it will be unfavorable for cost-effectiveness since a lower survival can be expected in those patients who are advised to discontinue ARPI treatment but do not adhere to this advice. On the other hand, second- or third-line treatments are more expensive than ARPI, which would be delayed in this situation.

The analyses were conducted from a Dutch healthcare perspective. However, costs and guidelines differ in other countries and regions and may thereby limit the extrapolation of our cost-effectiveness to other countries. In an extrapolation you need to consider the effect of a different price on the incremental costs, keeping in mind that both strategies contain the same treatment but some are more utilized in the ctDNA-guided treatment strategy. In addition, the WTP threshold may differ in other countries and settings. Therefore, it is important to review the acceptability curve as presented with specific WTP thresholds in mind. From this graph, it can be concluded that ctDNA-guided treatment decisions can only be cost-effective if WTP thresholds higher than €65,000 are adopted, being the mean ICER in our analysis.

## Conclusion

Multiple novel treatment options have been approved over the past years for the treatment of prostate cancer patients. Despite the significant benefits in health outcomes achieved by these treatments, the novel regimens may also cause a heavy burden on healthcare expenditures. ctDNA guidance in novel therapies and combination regimens might be used to evaluate early treatment response but more research is needed to determine whether this approach is cost-effective. In our study, we demonstrate that the use of ctDNA as a response marker after only 4 weeks of first-line ARPI treatment in mCRPC patients is likely cost-effective in the Dutch setting. This paves the way for integrating ctDNA-guided treatment optimization into the clinical management of mCRPC patients.

## Supplemental Material

sj-docx-1-tam-10.1177_17588359241305084 – Supplemental material for Circulating tumor DNA-guided treatment decision in metastatic castration-resistant prostate cancer patients: a cost-effectiveness analysis Supplemental material, sj-docx-1-tam-10.1177_17588359241305084 for Circulating tumor DNA-guided treatment decision in metastatic castration-resistant prostate cancer patients: a cost-effectiveness analysis by Catharina J. P. Op ’t Hoog, Sabien J. E. Bosman, Emmy Boerrigter, Niven Mehra, Inge M. van Oort, Nielka P. van Erp and Wietske Kievit in Therapeutic Advances in Medical Oncology

sj-docx-2-tam-10.1177_17588359241305084 – Supplemental material for Circulating tumor DNA-guided treatment decision in metastatic castration-resistant prostate cancer patients: a cost-effectiveness analysis Supplemental material, sj-docx-2-tam-10.1177_17588359241305084 for Circulating tumor DNA-guided treatment decision in metastatic castration-resistant prostate cancer patients: a cost-effectiveness analysis by Catharina J. P. Op ’t Hoog, Sabien J. E. Bosman, Emmy Boerrigter, Niven Mehra, Inge M. van Oort, Nielka P. van Erp and Wietske Kievit in Therapeutic Advances in Medical Oncology

sj-docx-3-tam-10.1177_17588359241305084 – Supplemental material for Circulating tumor DNA-guided treatment decision in metastatic castration-resistant prostate cancer patients: a cost-effectiveness analysis Supplemental material, sj-docx-3-tam-10.1177_17588359241305084 for Circulating tumor DNA-guided treatment decision in metastatic castration-resistant prostate cancer patients: a cost-effectiveness analysis by Catharina J. P. Op ’t Hoog, Sabien J. E. Bosman, Emmy Boerrigter, Niven Mehra, Inge M. van Oort, Nielka P. van Erp and Wietske Kievit in Therapeutic Advances in Medical Oncology

sj-pdf-4-tam-10.1177_17588359241305084 – Supplemental material for Circulating tumor DNA-guided treatment decision in metastatic castration-resistant prostate cancer patients: a cost-effectiveness analysis Supplemental material, sj-pdf-4-tam-10.1177_17588359241305084 for Circulating tumor DNA-guided treatment decision in metastatic castration-resistant prostate cancer patients: a cost-effectiveness analysis by Catharina J. P. Op ’t Hoog, Sabien J. E. Bosman, Emmy Boerrigter, Niven Mehra, Inge M. van Oort, Nielka P. van Erp and Wietske Kievit in Therapeutic Advances in Medical Oncology

## Declarations

**Ethics approval and consent to participate:** Not applicable.

**Consent for publication:** Not applicable.

**Availability of data and materials:** All used literature and data input for this study is included in the Supplemental Files. The generated datasets and models are available from the corresponding author on request.
