# Supplementary material for: Circulating tumor DNA-guided treatment decision in metastatic castration-resistant prostate cancer patients: a cost-effectiveness analysis
Source: Ther Adv Med Oncol. 2024 Dec 15;16:17588359241305084. doi: 10.1177/17588359241305084 (PMC11648017; doi:10.1177/17588359241305084)
Supplement: sj-docx-1-tam-10.1177_17588359241305084 – Supplemental material for Circulating tumor DNA-guided treatment decision in metastatic castration-resistant prostate cancer patients: a cost-effectiveness analysis [file sj-docx-1-tam-10.1177_17588359241305084.docx]

| Tyope | Parameter | Base value | Probabilistic | Deterministic | Standard error | Distribution (α, β) | Source |
| --- | --- | --- | --- | --- | --- | --- | --- |
| Discount rate | Costs | 0.040 | Fixed | 0.040 |  |  | (1) |
|  | Effects | 0.015 | Fixed | 0.015 |  |  | (1) |
| Transition probabilities  *Mortality* | Mortality enzalutamide | Cycle dependent |  |  |  |  | (2) |
|  | Mortality abiraterone | Cycle dependent |  |  |  |  | (3) |
|  | Mortality docetaxel 1^st^ line | Cycle dependent |  |  |  |  | (4) |
|  | Mortality cabazitaxel 2^nd^ line | Cycle dependent |  |  |  |  | (5) |
|  | Mortality cabazitaxel 3^rd^ line | Cycle dependent |  |  |  |  | (6) |
|  | Mortality BSC | Cycle dependent |  |  |  |  | (7) |
|  | Mortality docetaxel 2^nd^ line | Cycle dependent |  |  |  |  | (8) |
| *Progression* | PFS enzalutamide | Cycle dependent |  |  |  |  | (2) |
|  | PFS abiraterone | Cycle dependent |  |  |  |  | (9) |
|  | PFS docetaxel 1^st^ line | Cycle dependent |  |  |  |  | (4) |
|  | PFS cabazitaxel 2^nd^ line | Cycle dependent |  |  |  |  | (5) |
|  | PFS cabazitaxel 3^rd^ line | Cycle dependent |  |  |  |  | (6) |
|  | PFS docetaxel 2^nd^ line | Cycle dependent |  |  |  |  | (8) |
|  | Choice BSC after 1^st^ line | 0.280 | 0.272 | 0.280 | 0.01 | Beta (564.00, 1416.00) | (10) |
|  | Choice BSC after 2^nd^ line | 0.290 | 0.296 | 0.290 | 0.01 | Beta (288.00, 681.00) | (10) |
|  | Choice BSC after cabazitaxel | 0.000528 (per cycle, 15.2% in 18 mnd) | 0.001081 | 0.000528 (per cycle, 15.2% in 18 mnd) | 0.02 | Beta (57.90, 380.95) | (11) |
|  | Mapping algorithm utilities | - | - | - |  |  | (12) |
| Utility | Abiraterone | 0.804 | 0.650 | 0.804 | 0.131 | Beta (3.39, 1.80) | (13) |
|  | Enzalutamide | 0.788 | 0.744 | 0.788 | 0.129 | Beta (6.92, 2.13) | (14) |
|  | Docetaxel | 0.683 | 0.799 | 0.683 | 0.214 | Beta (2.23. 1.50) | (15) |
|  | Docetaxel scenario analysis | 0.796 |  | 0.796 | 0.130 | Beta (6.66, 1.96) | Mean utility enzalutamide and abiraterone |
|  | Cabazitaxel 2^nd^ line | 0.601 | 0.534 | 0.601 | 0.251 | Beta (1.28, 4.52) | (15) |
|  | Cabazitaxel 3^rd^ line | 0.628 | 0.835 | 0.628 | 0.242 | Beta (1.51, 1.49) | (16) |
|  | Best Supportive Care | 0.554 | 0.535 | 0.554 | 0.036 | Beta (103.34, 84.11) | (17) |
| Adverse events | Disutility febrile neutropenia | -0.210 |  |  |  |  | (18) |
|  | Disutility anemia | -0.119 |  |  |  |  | (19) |
|  | Disutility diarrhea | -0.212 |  |  |  |  | (19) |
|  | Body Surface Area | 1.91 | 1.905 | 1.91 | 0.01 | Gamma (140145.41, 0.00) | (20) |
|  | % patients with non-durable response abiraterone/enzalutamide | 0.85 | 0.836 | 0.85 | 0.07 | Beta (23.00, 4.00) | (21) |
| Costs  *Treatment* | Costs abiraterone_3w | €1983.66 | Fixed | €1983.66 |  |  | (22) |
|  | Costs enzalutamide_3w | €2268.00 | Fixed | €2268.00 |  |  | (22) |
|  | Costs docetaxel_3w | €548.60 | Fixed | €548.60 |  |  | (22) |
|  | Costs cabazitaxel_3w | €15087.32 | Fixed | €15087.32 |  |  | (22) |
|  | Costs daycare treatment | €357.70 | Fixed | €357.70 |  |  | (23) |
|  | Costs consult | €145.99 | Fixed | €145.99 |  |  | (23) |
|  | Costs CT | €214.92 | Fixed | €214.92 |  |  | (24) |
|  | Costs ctDNA-testing | €350 | Fixed | €350 |  |  |  |
|  | Costs BSC_3w | €2176.72 | €2299.10 | €2176.72 | 116.12 | Gamma (351.41, 6.19) | (25) |
| *Adverse Events* | Costs febrile neutropenia_3w | €3953.29 | €3836.26 | €3953.29 | 605.09 | Gamma (42.68, 96.62) | (26) |
|  | Costs anemia_3w | €87.06 | €85.60 | €87.06 | 87.06 | Gamma (57.32, 1.52) | (26) |
|  | Costs diarrhea_3w | €224.68 | €235.18 | €224.68 | 33.68 | Gamma (44.52, 5.05) | (26) |
|  | Inflation conversion to 2021 | - | - | - |  |  | (27) |
|  | % abiraterone in 1^st^ line | 0.5 | Fixed | 0.5 |  |  | Assumption |

**References**

1. Zorginstituut Nederland. Guideline for economic evaluations in healthcare. 2016.

2. Beer TM, Armstrong AJ, Rathkopf DE, Loriot Y, Sternberg CN, Higano CS, et al. Enzalutamide in metastatic prostate cancer before chemotherapy. N Engl J Med. 2014;371(5):424-33.

3. Ryan CJ, Smith MR, Fizazi K, Saad F, Mulders PF, Sternberg CN, et al. Abiraterone acetate plus prednisone versus placebo plus prednisone in chemotherapy-naive men with metastatic castration-resistant prostate cancer (COU-AA-302): final overall survival analysis of a randomised, double-blind, placebo-controlled phase 3 study. Lancet Oncol. 2015;16(2):152-60.

4. Oudard S, Fizazi K, Sengelov L, Daugaard G, Saad F, Hansen S, et al. Cabazitaxel Versus Docetaxel As First-Line Therapy for Patients With Metastatic Castration-Resistant Prostate Cancer: A Randomized Phase III Trial-FIRSTANA. J Clin Oncol. 2017;35(28):3189-97.

5. Eisenberger M, Hardy-Bessard AC, Kim CS, Geczi L, Ford D, Mourey L, et al. Phase III Study Comparing a Reduced Dose of Cabazitaxel (20 mg/m(2)) and the Currently Approved Dose (25 mg/m(2)) in Postdocetaxel Patients With Metastatic Castration-Resistant Prostate Cancer-PROSELICA. J Clin Oncol. 2017;35(28):3198-206.

6. de Wit R, de Bono J, Sternberg CN, Fizazi K, Tombal B, Wülfing C, et al. Cabazitaxel versus Abiraterone or Enzalutamide in Metastatic Prostate Cancer. N Engl J Med. 2019;381(26):2506-18.

7. Löffeler S, Weedon-Fekjaer H, Wang-Hansen MS, Sebakk K, Hamre H, Haug ES, Fosså SD. “Natural course” of disease in patients with metastatic castrate-resistant prostate cancer: Survival and prognostic factors without life-prolonging treatment. Scandinavian Journal of Urology. 2015;49(6):440-5.

8. Miyake H, Sugiyama T, Aki R, Matsushita Y, Tamura K, Motoyama D, et al. Comparison of Alternative Androgen Receptor-axis-targeted Agent (ARATA) and Docetaxel as Second-line Therapy for Patients With Metastatic Castration-resistant Prostate Cancer With Progression After Initial ARATA in Real-world Clinical Practice in Japan. Clin Genitourin Cancer. 2018;16(3):219-25.

9. Rathkopf DE, Smith MR, de Bono JS, Logothetis CJ, Shore ND, de Souza P, et al. Updated Interim Efficacy Analysis and Long-term Safety of Abiraterone Acetate in Metastatic Castration-resistant Prostate Cancer Patients Without Prior Chemotherapy (COU-AA-302). European Urology. 2014;66(5):815-25.

10. George DJ, Sartor O, Miller K, Saad F, Tombal B, Kalinovský J, et al. Treatment Patterns and Outcomes in Patients With Metastatic Castration-resistant Prostate Cancer in a Real-world Clinical Practice Setting in the United States. Clin Genitourin Cancer. 2020;18(4):284-94.

11. Rouyer M, Oudard S, Joly F, Fizazi K, Tubach F, Jove J, et al. Overall and progression-free survival with cabazitaxel in metastatic castration-resistant prostate cancer in routine clinical practice: the FUJI cohort. Br J Cancer. 2019;121(12):1001-8.

12. Diels J, Hamberg P, Ford D, Price PW, Spencer M, Dass RN. Mapping FACT-P to EQ-5D in a large cross-sectional study of metastatic castration-resistant prostate cancer patients. Qual Life Res. 2015;24(3):591-8.

13. Basch E, Autio K, Ryan CJ, Mulders P, Shore N, Kheoh T, et al. Abiraterone acetate plus prednisone versus prednisone alone in chemotherapy-naive men with metastatic castration-resistant prostate cancer: patient-reported outcome results of a randomised phase 3 trial. The Lancet Oncology. 2013;14(12):1193-9.

14. Loriot Y, Miller K, Sternberg CN, Fizazi K, De Bono JS, Chowdhury S, et al. Effect of enzalutamide on health-related quality of life, pain, and skeletal-related events in asymptomatic and minimally symptomatic, chemotherapy-naive patients with metastatic castration-resistant prostate cancer (PREVAIL): results from a randomised, phase 3 trial. The Lancet Oncology. 2015;16(5):509-21.

15. Thiery-Vuillemin A, Fizazi K, Sartor O, Oudard S, Bury D, Thangavelu K, et al. An analysis of health-related quality of life in the phase III PROSELICA and FIRSTANA studies assessing cabazitaxel in patients with metastatic castration-resistant prostate cancer. ESMO Open. 2021;6(2):100089.

16. Fizazi K, Kramer G, Eymard J-C, Sternberg CN, de Bono J, Castellano D, et al. Quality of life in patients with metastatic prostate cancer following treatment with cabazitaxel versus abiraterone or enzalutamide (CARD): an analysis of a randomised, multicentre, open-label, phase 4 study. The Lancet Oncology. 2020;21(11):1513-25.

17. Sandblom G, Carlsson P, Sennfält K, Varenhorst E. A population-based study of pain and quality of life during the year before death in men with prostate cancer. Br J Cancer. 2004;90(6):1163-8.

18. Gharaibeh M, McBride A, Bootman JL, Patel H, Abraham I. Economic evaluation for the US of nab-paclitaxel plus gemcitabine versus FOLFIRINOX versus gemcitabine in the treatment of metastatic pancreas cancer. Journal of Medical Economics. 2017;20(4):345-52.

19. Barqawi YK, Borrego ME, Roberts MH, Abraham I. Cost-effectiveness model of abiraterone plus prednisone, cabazitaxel plus prednisone and enzalutamide for visceral metastatic castration resistant prostate cancer therapy after docetaxel therapy resistance. Journal of Medical Economics. 2019;22(11):1202-9.

20. Sacco JJ, Botten J, Macbeth F, Bagust A, Clark P. The average body surface area of adult cancer patients in the UK: a multicentre retrospective study. PLoS One. 2010;5(1):e8933.

21. Verma S, Prajapati KS, Kushwaha PP, Shuaib M, Kumar Singh A, Kumar S, Gupta S. Resistance to second generation antiandrogens in prostate cancer: pathways and mechanisms. Cancer Drug Resist. 2020;3(4):742-61.

22. Medicijnkosten.nl: Zorginstituut Nederland; [Available from: <www.medicijnkosten.nl>.

23. Hakkaart-van Roijen L, Linden Nvd, Bouwmans C, Kanters T, Tan SS. Richtlijn voor het uitvoeren van economische evaluaties in de gezondheidszorg. Kostenhandleiding: methodologie van kostenonderzoek en referentieprijzen voor economische evaluaties in de gezondheidszorg. National Health Care Institute; 2016.

24. Passantenprijslijst: Isala; 2023 [Available from: <https://www.isala.nl/praktische-informatie/rekening-verzekering/passantenprijslijst/>.

25. Brinkman-Stoppelenburg A, Polinder S, Olij BF, van den Berg B, Gunnink N, Hendriks MP, et al. The association between palliative care team consultation and hospital costs for patients with advanced cancer: An observational study in 12 Dutch hospitals. Eur J Cancer Care (Engl). 2020;29(3):e13198.

26. Peters ML, de Meijer C, Wyndaele D, Noordzij W, Leliveld-Kors AM, van den Bosch J, et al. Dutch Economic Value of Radium-223 in Metastatic Castration-Resistant Prostate Cancer. Appl Health Econ Health Policy. 2018;16(1):133-43.

27. Jaarmutatie consumentenprijsindex: Centraal Bureau voor Statistiek (CBS); 2022 [Available from: <https://opendata.cbs.nl/#/CBS/nl/dataset/70936ned/table?searchKeywords=consumentenprijsindex>.
