# Supplementary material for: Circulating tumor DNA-guided treatment decision in metastatic castration-resistant prostate cancer patients: a cost-effectiveness analysis
Source: Ther Adv Med Oncol. 2024 Dec 15;16:17588359241305084. doi: 10.1177/17588359241305084 (PMC11648017; doi:10.1177/17588359241305084)
Supplement: sj-docx-2-tam-10.1177_17588359241305084 – Supplemental material for Circulating tumor DNA-guided treatment decision in metastatic castration-resistant prostate cancer patients: a cost-effectiveness analysis [file sj-docx-2-tam-10.1177_17588359241305084.docx]

Supplementary file 2. Overview results sensitivity analyses.

| Analysis | iNMB (€) |
| --- | --- |
| Base Case | 5277.22 |
| Abiraterone Discount: 50% | 7336,197 |
| Abiraterone Discount: 80% | 8571.585 |
| Abiraterone Discount: 90% | 8983.381 |
| Enzalutamide Discount: 50% | 7631.334 |
| Enzalutamide Discount: 80% | 9043.803 |
| Enzalutamide Discount: 90% | 9514.627 |
| Cabazitaxel Discount: 50% | 6425.188 |
| Cabazitaxel Discount: 80% | 6789.002 |
| Cabazitaxel Discount: 90% | 6910.273 |
| Cost of ctDNA €300 | 5427.10 |
| Cost of ctDNA €200 | 5726.87 |
| Cost of ctDNA €100 | 6026.63 |
| Proportion of Abiraterone 1 | 48.747 |
| Proportion of Abiraterone 0 | 6968.349 |
| Prediction value of ctDNA 0.95 | 5213.499 |
| Prediction value of ctDNA 0.75 | 5340.937 |
| Prediction value of ctDNA 0.65 | 5404.656 |
| Abiraterone/Enzalutamide registration trial data | -1867.966 |
| Cabazitaxel PROSELICA trial data | 5058.158 |
| Utility docetaxel scenario analysis | 5495.077 |
| Second-line doxetaxel data | 4809.289 |
|  | |
