# Supplementary material for: Circulating tumor DNA-guided treatment decision in metastatic castration-resistant prostate cancer patients: a cost-effectiveness analysis
Source: Ther Adv Med Oncol. 2024 Dec 15;16:17588359241305084. doi: 10.1177/17588359241305084 (PMC11648017; doi:10.1177/17588359241305084)
Supplement: sj-docx-3-tam-10.1177_17588359241305084 – Supplemental material for Circulating tumor DNA-guided treatment decision in metastatic castration-resistant prostate cancer patients: a cost-effectiveness analysis [file sj-docx-3-tam-10.1177_17588359241305084.docx]

| **Citation of published trial** | **ClinicalTrials.gov Identifier** | **Acronym** | **Start Date** | **End Date** | **Sample Size** | **Researched drug** | **Comparison** | **Stage of prostate cancer** | **Line of treatment** | **Included/excluded** | **Model** | **Notes** |
| --- | --- | --- | --- | --- | --- | --- | --- | --- | --- | --- | --- | --- |
| Armstrong AJ, Szmulewitz RZ, Petrylak DP, Holzbeierlein J, Villers A, Azad A, et al. ARCHES: A Randomized, Phase III Study of Androgen Deprivation Therapy With Enzalutamide or Placebo in Men With Metastatic Hormone-Sensitive Prostate Cancer. J Clin Oncol. 2019;37(32):2974-86. | NCT02677896 | ARCHES | 2016 | 2018 | 1150 | Enzalutamide | Placebo | mHSPC | First-/Second-line | Excluded | - | In mHSPC patients |
| Beer TM, Armstrong AJ, Rathkopf DE, Loriot Y, Sternberg CN, Higano CS, et al. Enzalutamide in metastatic prostate cancer before chemotherapy. N Engl J Med. 2014;371(5):424-33. | NCT01212991 | PREVAIL | 2010 | 2014 | 1717 | Enzalutamide | Placebo + prednison | Chemotherapy-naïve mCRPC | First-line | Included | Base model - standard of care arm Scenario analysis - both arms | Most representative for our study population |
| Bono JSd, Logothetis CJ, Molina A, Fizazi K, North S, Chu L, et al. Abiraterone and Increased Survival in Metastatic Prostate Cancer. New England Journal of Medicine. 2011;364(21):1995-2005. | NCT00638690 | COU-AA-301 | 2008 | 2010 | 1195 | Abiraterone + prednisone | Placebo + prednisone | Post-docetaxel mCRPC | Second-line | Excluded | - | Abiraterone not as first-line treatment |
| de Bono JS, Oudard S, Ozguroglu M, Hansen S, Machiels J-P, Kocak I, et al. Prednisone plus cabazitaxel or mitoxantrone for metastatic castration-resistant prostate cancer progressing after docetaxel treatment: a randomised open-label trial. The Lancet. 2010;376(9747):1147-54. | NCT00417079 | TROPIC | 2007 | 2009 | 755 | Cabazitaxel + prednisone | Mitoxantrone | mCRPC | Second-line | Excluded | - | Comparison with mitoxantrone |
| de Wit R, de Bono J, Sternberg CN, Fizazi K, Tombal B, Wülfing C, et al. Cabazitaxel versus Abiraterone or Enzalutamide in Metastatic Prostate Cancer. N Engl J Med. 2019;381(26):2506-18. | NCT02485691 | CARD | 2015 | 2018 | 255 | Cabazitaxel | Abiraterone or enzalutamide | Post-docetaxel and abi/enza mCRPC | Third-line | Included | Base model - both arms | Most representative for our study population since cabazitaxel is used as third-line after docetaxel and abi/enza |
| Eisenberger M, Hardy-Bessard AC, Kim CS, Geczi L, Ford D, Mourey L, et al. Phase III Study Comparing a Reduced Dose of Cabazitaxel (20 mg/m(2)) and the Currently Approved Dose (25 mg/m(2)) in Postdocetaxel Patients With Metastatic Castration-Resistant Prostate Cancer-PROSELICA. J Clin Oncol. 2017;35(28):3198-206. | NCT01308580 | PROSELICA | 2011 | 2015 | 1200 | Cabazitaxel 20 | Cabazitaxel 25 | mCRPC | Second-line | Included | Scenario analysis - intervention arm | Cabazitaxel can be seen as second-line after docetaxel if patients only use ARPI for 4 weeks |
| Fizazi K, Foulon S, Carles J, Roubaud G, McDermott R, Fléchon A, et al. Abiraterone plus prednisone added to androgen deprivation therapy and docetaxel in de novo metastatic castration-sensitive prostate cancer (PEACE-1): a multicentre, open-label, randomised, phase 3 study with a 2x2 factorial design. The Lancet. 2022;399(10336):1695-707. | NCT01957436 | PEACE-1 | 2013 | 2018 | 1173 | Abiraterone + prednisone | Docetaxel | mHSPC | First-line | Excluded | - | In mHSPC patients |
| Fizazi K, Tran N, Fein L, Matsubara N, Rodriguez-Antolin A, Alekseev BY, et al. Abiraterone plus Prednisone in Metastatic, Castration-Sensitive Prostate Cancer. New England Journal of Medicine. 2017;377(4):352-60. | NCT01715285 | LATITUDE | 2013 | 2014 | 1119 | ADT + abiraterone + prednisone | ADT + placebo | High risk mHSPC | First-line | Excluded | - | In mHSPC patients |
| Miyake H, Sugiyama T, Aki R, Matsushita Y, Tamura K, Motoyama D, et al. Comparison of Alternative Androgen Receptor-axis-targeted Agent (ARATA) and Docetaxel as Second-line Therapy for Patients With Metastatic Castration-resistant Prostate Cancer With Progression After Initial ARATA in Real-world Clinical Practice in Japan. Clin Genitourin Cancer. 2018;16(3):219-25. | - | - | 2014 | 2016 | 222 | Abiraterone or enzalutamide | Docetaxel | mCRPC | Second-line | Included | Scenario analysis - both arms | Only available study investigating docetaxel as second-line treatment |
| Oudard S, Fizazi K, Sengelov L, Daugaard G, Saad F, Hansen S, et al. Cabazitaxel Versus Docetaxel As First-Line Therapy for Patients With Metastatic Castration-Resistant Prostate Cancer: A Randomized Phase III Trial-FIRSTANA. J Clin Oncol. 2017;35(28):3189-97. | NCT01308567 | FIRSTANA | 2011 | 2018 | 1168 | Cabazitaxel | Docetaxel | Chemotherapy-naïve mCRPC | First-line | Included | Base model - both arms | Most representative for our study population. Docetaxel given as first-line treatment. No research is available of docetaxel after ARPI as second-line |
| Quinn DI, Tangen CM, Hussain M, Lara PN, Jr., Goldkorn A, Moinpour CM, et al. Docetaxel and atrasentan versus docetaxel and placebo for men with advanced castration-resistant prostate cancer (SWOG S0421): a randomised phase 3 trial. Lancet Oncol. 2013;14(9):893-900. | NCT00134056 | SWOG S0421 | 2006 | 2016 | 994 | Docetaxel + prednisone | Docetaxel + prednisone + atrasentan | mCRPC | Frist-line | Excluded | - | Comparison with artrasentan |
| Rathkopf DE, Smith MR, de Bono JS, Logothetis CJ, Shore ND, de Souza P, Fizazi K, Mulders PF, Mainwaring P, Hainsworth JD, Beer TM, North S, Fradet Y, Van Poppel H, Carles J, Flaig TW, Efstathiou E, Yu EY, Higano CS, Taplin ME, Griffin TW, Todd MB, Yu MK, Park YC, Kheoh T, Small EJ, Scher HI, Molina A, Ryan CJ, Saad F. Updated interim efficacy analysis and long-term safety of abiraterone acetate in metastatic castration-resistant prostate cancer patients without prior chemotherapy (COU-AA-302). Eur Urol. 2014 Nov;66(5):815-25. doi: 10.1016/j.eururo.2014.02.056. | NCT00887198 | COU-AA-302 | 2009 | 2014 | 1088 | Abiraterone + prednisone | Placebo + prednisone | Chemotherapy-naïve mCRPC | First-line | Included | Base model - standard of care arm Scenario analysis - both arms | Updated analaysis COU-AA-302. Most representative for our study population |
| Ryan CJ, Smith MR, Fizazi K, Saad F, Mulders PF, Sternberg CN, et al. Abiraterone acetate plus prednisone versus placebo plus prednisone in chemotherapy-naive men with metastatic castration-resistant prostate cancer (COU-AA-302): final overall survival analysis of a randomised, double-blind, placebo-controlled phase 3 study. Lancet Oncol. 2015;16(2):152-60. | NCT00887198 | COU-AA-302 | 2009 | 2014 | 1088 | Abiraterone + prednisone | Placebo + prednisone | Chemotherapy-naïve mCRPC | First-line | Included | Base model - standard of care arm Scenario analysis - both arms | Most representative for our study population |
| Scher HI, Fizazi K, Saad F, Taplin M-E, Sternberg CN, Miller K, et al. Increased Survival with Enzalutamide in Prostate Cancer after Chemotherapy. New England Journal of Medicine. 2012;367(13):1187-97. | NCT00974311 | AFFIRM | 2009 | 2011 | 1199 | Enzalutamide | Placebo | Post-docetaxel mCRPC | Second-/Third-line | Excluded | - | Enzalutamide not as first-line treatment |
| Sweeney CJ, Chen Y-H, Carducci M, Liu G, Jarrard DF, Eisenberger M, et al. Chemohormonal Therapy in Metastatic Hormone-Sensitive Prostate Cancer. New England Journal of Medicine. 2015;373(8):737-46. | NCT00309985 | CHAARTED | 2006 | 2012 | 790 | ADT + docetaxel | ADT | mHSPC | Frist-line | Excluded | - | In mHSPC patients |
| Tannock IF, Wit Rd, Berry WR, Horti J, Pluzanska A, Chi KN, et al. Docetaxel plus Prednisone or Mitoxantrone plus Prednisone for Advanced Prostate Cancer. New England Journal of Medicine. 2004;351(15):1502-12. | NCT00675545 | TAX327 | 2000 | 2002 | 1006 | Docetaxel + prednisone | Mitoxantrone | mCRPC | Second-line | Excluded | - | Comparison to Mitoxantrone |
| Tolmeijer SH, Boerrigter E, Sumiyoshi T, Kwan EM, Ng SWS, Annala M, et al. Early On-treatment Changes in Circulating Tumor DNA Fraction and Response to Enzalutamide or Abiraterone in Metastatic Castration-Resistant Prostate Cancer. Clin Cancer Res. 2023;29(15):2835-44. | NCT02426333; NCT02471469 | REFINE | 2015 | 2018 | 81 | Abiraterone or enzalutamide | Early switch | mCRPC | First-/Second-line | Included | Base model - intervention arm Scenario analysis - intervention arm | The cost-effectiveness analysis was based on this study |
