# Supplementary material for: Circulating tumor DNA-guided treatment decision in metastatic castration-resistant prostate cancer patients: a cost-effectiveness analysis
Source: Ther Adv Med Oncol. 2024 Dec 15;16:17588359241305084. doi: 10.1177/17588359241305084 (PMC11648017; doi:10.1177/17588359241305084)
Supplement: sj-pdf-4-tam-10.1177_17588359241305084 – Supplemental material for Circulating tumor DNA-guided treatment decision in metastatic castration-resistant prostate cancer patients: a cost-effectiveness analysis [file sj-pdf-4-tam-10.1177_17588359241305084.pdf]

CHEERS 2022 Checklist

**Title**			
Title	1	Identify the study as an economic evaluation and specify the interventions being compared.	Title, Abstract, Page 4
**Abstract**			
Abstract	2	Provide a structured summary that highlights context, key methods, results, and alternative analyses.	Abstract, Page 4
**Introduction**			
Background and objectives	3	Give the context for the study, the study question, and its practical relevance for decision making in policy or practice.	Introduction, Third Paragraph
Methods			
**Health economic analysis plan**	**4**	**Indicate whether a health economic analysis plan was developed and where available.**	**Methods, First Paragraph**
Study population	5	Describe characteristics of the study population (such as age range, demographics, socioeconomic, or clinical characteristics).	Methods, First Paragraph
Setting and location	6	Provide relevant contextual information that may influence findings.	Methods, Third Paragraph
Comparators	7	Describe the interventions or strategies being compared and why chosen.	Methods, First Paragraph
Perspective	8	State the perspective(s) adopted by the study and why chosen.	Methods, First Paragraph
Time horizon	9	State the time horizon for the study and why appropriate.	Methods, First Paragraph

(continued)

Discount rate	10	Report the discount rate(s) and reason chosen.	Methods, Fourth Paragraph
Selection of outcomes	11	Describe what outcomes were used as the measure(s) of benefit(s) and harm(s).	Methods, Fourth Paragraph
Measurement of outcomes	12	Describe how outcomes used to capture benefit(s) and harm(s) were measured.	Methods, Sixth Paragraph
Valuation of outcomes	13	Describe the population and methods used to measure and value outcomes.	Methods, Sixth Paragraph
Measurement and valuation of resources and costs	14	Describe how costs were valued.	Methods, Model data input
Currency, price date, and conversion	15	Report the dates of the estimated resource quantities and unit costs, plus the currency and year of conversion.	Methods, Model data input
Rationale and description of model	16	If modelling is used, describe in detail and why used. Report if the model is publicly available and where it can be accessed.	Methods, Model overview
Analytics and assumptions	17	Describe any methods for analysing or statistically transforming data, any extrapolation methods, and approaches for validating any model used.	Methods , Analysis, Appendix
Characterising heterogeneity	18	Describe any methods used for estimating how the results of the study vary for subgroups.	Methods , Last two subheadings
Characterising distributional effects	19	Describe how impacts are distributed across different individuals or adjustments made to reflect priority populations.	Methods , Analysis

(continued)

Characterising uncertainty	20	Describe methods to characterise any sources of uncertainty in the analysis.	Methods, Last two subheadings
Approach to engagement with patients and others affected by the study	21	Describe any approaches to engage patients or service recipients, the general public, communities, or stakeholders (such as clinicians or payers) in the design of the study.	Not applicable
**Results**			
Study parameters	22	Report all analytic inputs (such as values, ranges, references) including uncertainty or distributional assumptions.	Results, First Paragraph, Appendix
Summary of main results	23	Report the mean values for the main categories of costs and outcomes of interest and summarise them in the most appropriate overall measure.	Results, First Paragraph, Appendix
Effect of uncertainty	24	Describe how uncertainty about analytic judgments, inputs, or projections affect findings. Report the effect of choice of discount rate and time horizon, if applicable.	Results, Sensitivy and Scenario analysis
Effect of engagement with patients and others affected by the study	25	Report on any difference patient/service recipient, general public, community, or stakeholder involvement made to the approach or findings of the study	Not applicable
Discussion			

(continued)

Study findings, limitations, generalisability, and current knowledge	26	Report key findings, limitations, ethical or equity considerations not captured, and how these could affect patients, policy, or practice.	Discussion
Other relevant information			
Source of funding	27	Describe how the study was funded and any role of the funder in the identification, design, conduct, and reporting of the analysis	End of manuscript
Conflicts of interest	28	Report authors conflicts of interest according to journal or International Committee of Medical Journal Editors requirements.	End of manuscript

From: Husereau D, Drummond M, Augustovski F, et al. Consolidated Health Economic Evaluation Reporting Standards 2022 (CHEERS 2022) Explanation and Elaboration: A Report of the ISPOR CHEERS II Good Practices Task Force. Value Health 2022;25. doi:10.1016/j.jval.2021.10.008
